# A Comparative Analysis of Clinical Outcomes between Proximal and Distal Recipient Vessel Microanastomosis for Lower Limb Reconstruction

**DOI:** 10.1055/s-0045-1811169

**Published:** 2025-09-25

**Authors:** Ebenezer J. C. Asirvatham, Bala Prasanth J, Atul Philipose, Jonathan Victor, Ashish Kumar Gupta, Shashank Lamba

**Affiliations:** 1 Department of Plastic and Reconstructive Surgery, Christian Medical College, Vellore, Tamil Nadu, India

**Keywords:** lower limb reconstruction, recipient vessels, distal versus proximal, anastomosis

## Abstract

**Background:**

This study aims to compare the clinical outcomes of lower limb free-flap reconstruction with vascular anastomosis performed proximal versus distal to the zone of injury. Very few comparative studies on this topic have been published to date. These studies are discussed in this article.

**Materials and Methods:**

A retrospective analysis of microvascular free-flap reconstructions for lower extremity defects over a 5-year period, from 2018 to 2023, was conducted. Outcomes of vascular anastomosis distal to the zone of injury were compared with those proximal to the zone of injury. Clinical parameters evaluated included flap failure, arterial and venous compromise, operative takebacks, and their success rates.

**Results:**

Of the 101 cases included in our analysis, 81 underwent vascular anastomosis proximal to the zone of injury, and 20 underwent distal anastomosis. In total, 72 (88.88%) proximal and 18 (90%) distal anastomoses were successful. Arterial thrombus occurred in 1 case (5%) among those who underwent distal anastomosis and in 4 cases (4.9%) of the proximal anastomosis cohort, whereas venous thrombus was seen in 1 case (5%) of the distal group and 12 cases (14.81%) of the proximal group. No statistically significant differences were found in clinical outcomes between the groups.

**Conclusion:**

Free tissue transfer using recipient vessels distal to the zone of injury is a reliable option for lower limb reconstruction. Distal anastomosis, which can be technically less challenging, demonstrates survival rates comparable to proximal anastomosis and can be considered a viable approach in selected patients.

## Introduction

Since the advent of limb salvage surgeries, microvascular free tissue transfer has commonly been preferred as the first choice for lower limb reconstruction. 1 Compared to other regions, entire circumferential segment of the lower extremity is often damaged in high-energy trauma. 2 3 This zone of injury is known to extend beyond what is macroscopically visible and failure to recognize the true extent of this thrombogenic zone may lead to flap failure. 2 3 Flap failure is frequently attributed to problems involving the vascular anastomosis or injuries to the recipient vessels. 4 Most surgeons prefer to do the anastomosis proximal to the zone of injury to avoid the theoretical risks associated with blood flow traversing damaged tissue. 3 It has been assumed that unfavorable changes in the vessel quality, such as caliber alterations within and distal to the trauma site, may result in flap failure. 2 3 However, performing proximal anastomosis is not always feasible, as the vessels lie deep under the muscles, and performing end-to-side anastomosis in a narrow and deep space can be challenging. In contrast, sites for distal anastomosis involving the anterior and posterior tibial arteries are more easily accessible. 3

Studies have demonstrated successful outcomes in microvascular free-flap reconstruction of the lower extremity using recipient vessels distal to the zone of injury. 4 5 6 This study aims to compare the outcomes of anastomoses performed distal versus proximal to the zone of injury in lower limb reconstruction.

## Materials and Methods

After obtaining clearance from the institutional review board (IRB No. 15990), we retrospectively reviewed the records of all patients who underwent lower extremity reconstruction with microvascular free flaps at our institute between January 2018 and November 2023. Data collected included age, gender, type of injury based on the Gustilo classification, composition of the free flap, time between injury and reconstruction, whether recipient vessels were proximal or distal to the zone of injury, and postoperative outcomes such as flap failure, operative takebacks, and complications.

Total flap failure was defined as flap compromise requiring complete debridement and either coverage with another flap or limb amputation. Partial flap failure was defined as flap-related complications requiring additional surgical procedures, such as wound breakdown management or flap debridement, within the first 3 months after the initial free-flap coverage.

Preoperatively, all patients were examined clinically. A computed tomography angiogram was performed in selected cases where the peripheral pulses were not palpable.

**Fig. 1:**
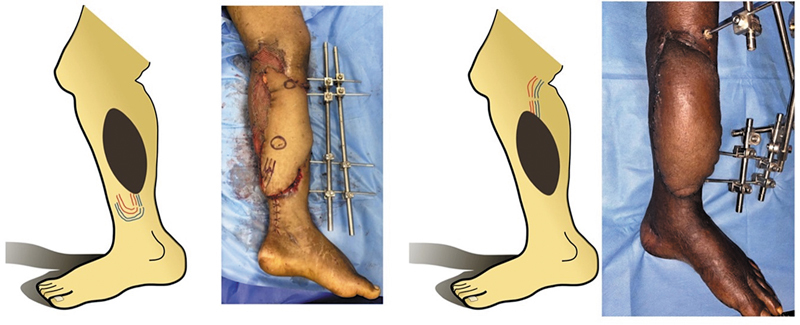
Diagrammatic representation and clinical picture of distal (right) and proximal (left) anastomosis.

Intraoperatively, following the dissection of the recipient vessels (proximal/distal; Fig. 1 ) and confirmation of adequate flow, the flap was harvested. After completion of the microvascular anastomosis, heparin was administered intraoperatively only in cases that required revision of the anastomosis due to arterial or venous thrombosis.

Postoperatively, free flap monitoring was performed through serial clinical examinations—hourly on the first postoperative day and every 4 hours on the second day. Patients received low-molecular-weight heparin or unfractionated heparin as part of deep vein thrombosis prophylaxis, and antibiotics were administered based on culture and sensitivity results. Compression bandaging and limb dangling were initiated on the fifth postoperative day. Ambulation was started based on orthopaedic recommendations.

Patient demographics and outcome variables were reported using standard summary statistics and compared using the chi-squared test or Fisher's exact test, as appropriate. A *p* -value of  < 0.05 was considered statistically significant. All analyses were performed using SPSS Statistics Version 21.0 (IBM Corporation, Chicago, Illinois, United States).

**Fig. 2:**
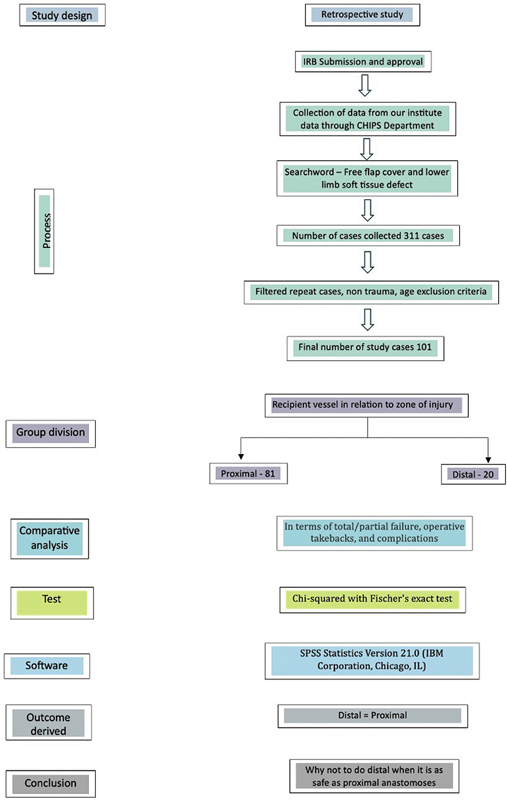
Flowchart of the full study process.

The study process is simplified and illustrated as a flow chart in Fig. 2 , and a diagrammatic representation, along with clinical images, is provided in Fig. 1 .

## Results

### Demographics

The median age in the distal group was 31 years, and in the proximal group, it was 34 years.

**Fig. 3:**
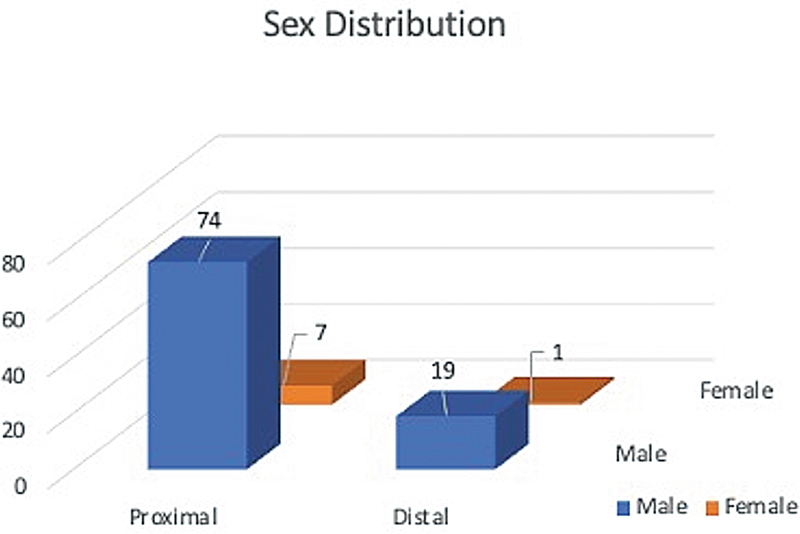
Sex distribution of the cases.

The male-to-female ratio was 19 (95%) to 1 (5%) in the distal group and 74 (91.36%) to 7 (8.64%) in the proximal group, as shown in Fig. 3 .

### Case Distribution

**Fig. 4:**
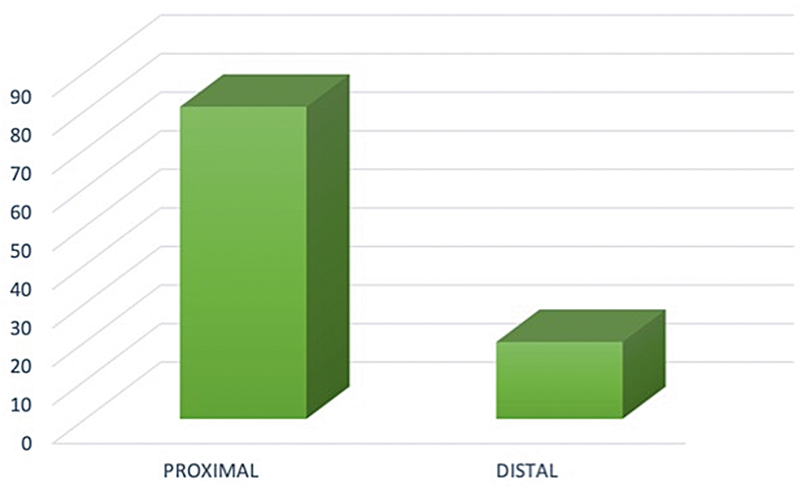
Number of proximal and distal cases.

Of the 101 cases included in our analysis, 81 (80.19%) underwent vascular anastomosis proximal to the zone of injury, and 20 (19.8%) underwent anastomosis distal to the zone of injury, as shown in Fig. 4 .

### Location of the Defect

**Fig. 5:**
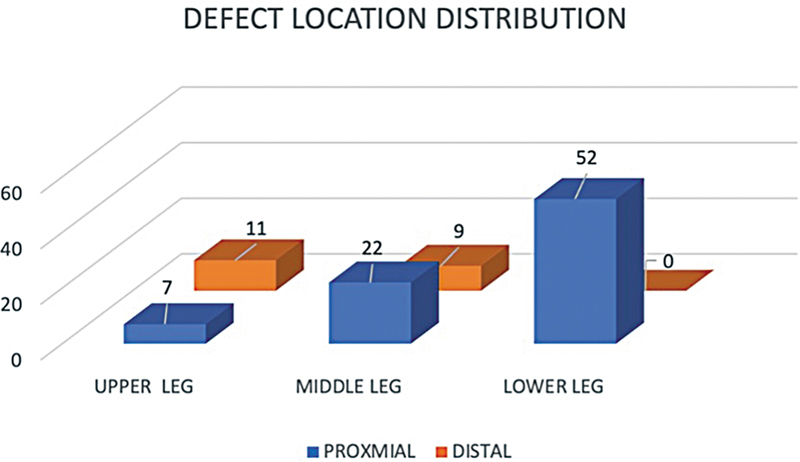
Distribution of the cases based on defect location.

Most of the defects were located in the upper and middle third of the leg in the group where anastomosis was performed distal to the zone of injury ( Fig. 5 ).

The majority of cases were classified as Gustilo type IIIB and IIIC. Distal anastomosis was performed in one case of type IIIC injury using an end-to-side technique.

**Fig. 6:**
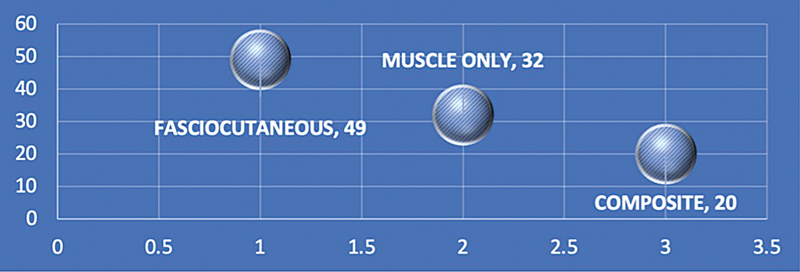
Flap composition of proximal and distal cases combined.

**Fig. 7:**
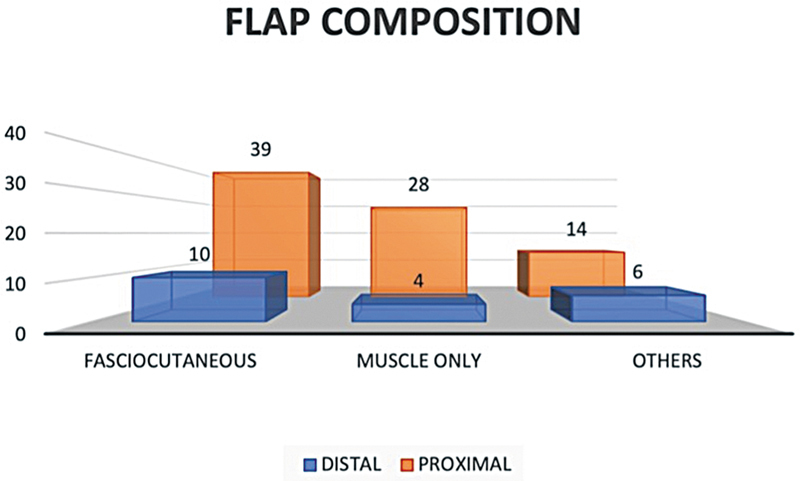
Distribution of cases based on flap composition in each group.

Of the 101 free flaps, 49 were fasciocutaneous, 32 were muscle-only, and the remaining 20 were composite flaps, as shown in Fig. 6 . The distribution of flap types within each group (i.e., proximal vs. distal) is shown in Fig. 7 .

### Time Distribution

**Fig. 8:**
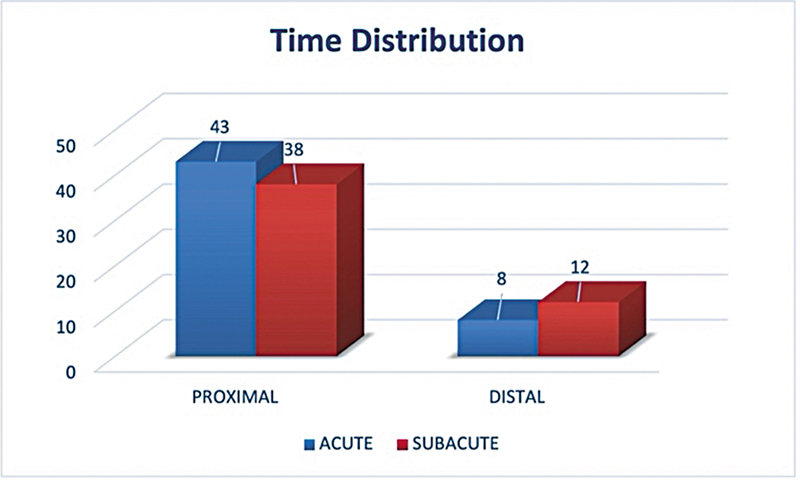
Time distribution.

Anastomoses of 8 cases (40%) in the distal group and 43 cases (53.08%) in the proximal group were performed during the acute period (<1 week). In addition, 12 cases (60%) in the distal group and 38 cases (46.91%) in the proximal group were performed during the subacute period (1–9 weeks), as shown in Fig. 8 .

### Recipient Vessels

**Fig. 9:**
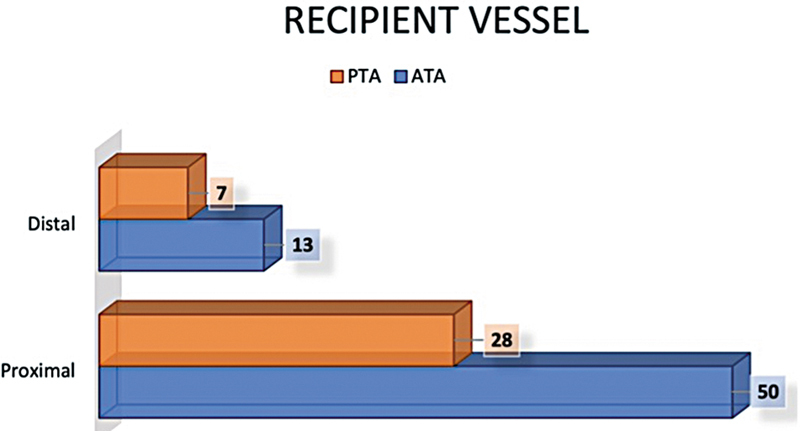
Number of case distribution based on recipient vessel.

Most anastomoses were performed using the anterior tibial artery, as shown in Fig. 9 . No significant difference was noted in outcomes based on the choice of recipient vessel (anterior or posterior tibial artery) or the type of anastomosis (end-to-end or end-to-side). In cases where the anastomosis was performed distal to the zone of injury, the majority (15 cases) were done in an end-to-end fashion. End-to-side anastomosis was performed in 5 cases in the distal group and 17 cases in the proximal group.

### Results and Comparison

Microvascular anastomosis was successful in 72 out of 81 proximal cases (88.88%) and in 18 out of 20 distal cases (90%) as shown in Table 1 . Of the two flap failures in the distal group, one required amputation and the other was managed with local flap. Of the nine flap failures in the proximal group, six were managed with local flap and three with skin grafts.

**Table 1:** Results of both groups in each variable

Variable	Distal		Proximal		*p-* Value	*N*
	Mean ± SD	Median (IQR)	Mean + SD	Median (IQR)		
**Age**	36.90 ± 15.44	31.50 (25.50)	34.05 ± 15.47	34 (21.44)	0.462	101
**Variables**		**Subdivision**	**Recipient vessel**			
			**Distal**	**Proximal**	***p-*** **Value**	***N***
			***n*** **(%)**	***n*** **(%)**		
**Sex**		Male	19 (95)	74 (91.36)	1.000	101
		Female	1 (5)	7 (8.64)		
**Gustilo**		3B	18 (94.74)	65 (89.04)	0.679	90
		3C	1 (5.26)	8 (10.96)		
**Flap composition**		Fasciocutaneous	10 (71.43)	39 (58.21)	0.349	81
		Muscle only	4 (28.57)	28 (41.79)		
**Time interval between injury and flap days**		Acute	8 (40)	43 (53.08)	0.413	101
		Subacute	12 (60)	38 (46.91)		
**Partial flap failure**		Yes	1 (14.29)	1 (3.57)	0.376	101
		No	6 (85.71)	27 (96.43)		
**Total flap failure**		Yes	2 (10)	9 (11.11)	1.000	101
		No	18 (90)	72 (88.89)		
**Arterial complication**		Yes	1 (50)	3 (18.75)	0.405	18
		No	1 (50)	13 (81.25)		
**Venous complication**		Yes	1 (33.33)	14 (77.78)	0.184	21
		No	2 (66.67)	4 (22.22)		
**Operative takeback**		Yes	1 (5)	13 (16.05)	0.291	101
		No	19 (95)	68 (83.95)		
**Success after operative takeback**		Yes	0 (0)	7 (53.80)	1.000	14
		No	1 (100)	6 (46.20)		

Abbreviation: SD, standard deviation.

Arterial thrombus occurred in one patient (5%) in the distal anastomosis group and in four patients (4.9%) in the proximal group. Venous thrombus was observed in 1 patient (5%) in the distal group and in 12 patients (14.81%) in the proximal group.

There was 1 operative takeback (5%) among the 20 distal anastomosis cases, compared to 13 takebacks (16.05%) among the 81 proximal cases. Of the 13 takebacks, 7 (53.80%) were successful. One flap could not be salvaged in the distal anastomosis group.

**Fig. 10:**
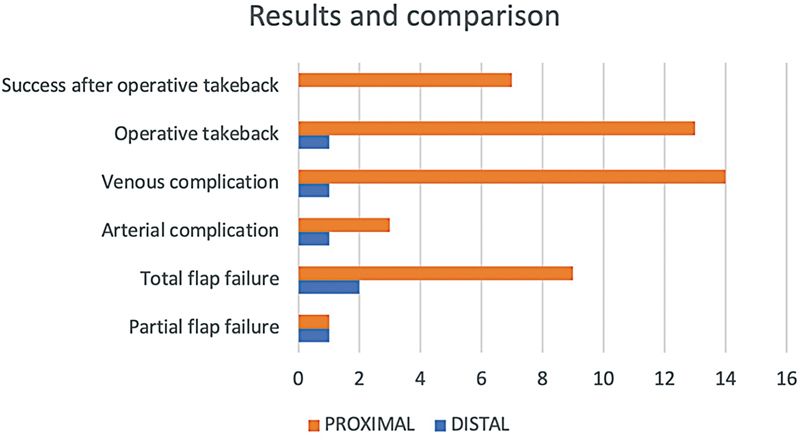
Overall result comparison between proximal and distal groups.

No statistically significant difference in clinical outcomes was observed between the two groups, as summarized in Table 1 and Fig. 10 .

All the cases of distal anastomosis were performed by a single experienced surgeon with 8 years of microvascular experience. In contrast, proximal anastomoses were performed by surgeons with varied levels of experience (ranging from 3 to 20 years, with a mean of 7.2 years).

## Discussion

Lower limb salvage with microvascular free tissue transfer has the highest flap loss rates—ranging from 4 to 20%—compared to any other anatomical site. 7 8 9 Multiple factors influence flap survival, including the severity of injury, size of the defect, recipient vessel selection, surgeon experience, and the timing of surgery. These factors are interdependent and can produce varied outcomes. 3 9

The vast majority of flaps in lower limb reconstruction are traditionally anastomosed to proximal recipient vessels, with distal sites being the least preferred. The debate over the optimal site for recipient vessels remains unresolved. 10 This study aimed to audit and analyze the impact of using distal recipient vessels on free flap outcomes in our population.

Godina was among the first to evaluate the timing of surgery in relation to recipient vessels. He recommended early defect coverage—within the first 3 days post-injury—after adequate debridement, in the absence of scarring. He observed a zone of inflammation extending more than 10 cm proximal to the wound in cases operated after the third day and thus advised using proximal vessels beyond this zone, while avoiding previously divided vessels. 7

The concept of the “Zone of Injury,” introduced in the 1980s, aimed to integrate the effects of inflammation and time on vessel selection. 11 However, Isenberg and Sherman later challenged this concept by successfully performing anastomoses within 6 cm of the wound edge, without extensive proximal dissection. 12 Loos et al attempted to objectively define this nebulous zone, 10 as many authors had reported up to 90% success using vessels within or distal to it. 4 5 13 14 Nonetheless, its borders remain subjective, varying by patient and injury extent.

Recent studies have confirmed the feasibility of distal anastomosis in severe wounds, such as Gustilo–Anderson type IIIB injuries, which constitute the majority of microsurgical cases. 15 16 17 Stranix et al, in a systematic review, found similar patency and survival rates between proximal and distal anastomoses. 15 Most retrospective studies in that review showed an 80:20 preference for proximal over distal anastomoses. Failure rates were approximately 7.6% for proximal and 9.5% for distal anastomoses. In their own audit, the failures rates were equal at 9.3%, with a 4:1 ratio of proximal to distal cases (252:60). Interestingly, arterial compromise was more frequent in the proximal group, while venous thrombosis was more common in the distal group. Similar findings were reported by a study from Oxford, which included a separate arm for vessels located directly within the wound. 18

Hallock proposed eight criteria for selecting proximal recipient vessels, based on poor outcomes with distal recipient sites. 11 19 He advised using distal vessels only when proximal options or vein grafts were unavailable. In contrast, the Pennsylvania group allows the use of distal or in-zone vessels at the surgeon's discretion. 20

Several algorithms have since been developed using pre- and intra-operative criteria, some even considering perforators as potential recipient vessels. We suggest that, rather than assessing the zone of injury as a two-dimensional area, evaluating it as a three-dimensional block provides a more accurate and practical framework for selecting recipient vessels for anastomosis. 10 14 19 21 22 23 24

Preoperative contraindications for distal recipient vessel use include clear evidence of thrombosis within or near the injury zone. A large-scale study from Taiwan recommended thorough clinical examination and the use of a handheld Doppler. In the acute setting, the vessel closest to the wound was dissected retrograde into healthy tissue. In delayed cases, antegrade dissection was performed from a normal area toward the wound. Importantly, no difference in failure rates was observed between previously occluded (but dissected to healthy) vessels and normal vessels. A simple spurt test was often sufficient to assess arterial patency. In severe cases, vein grafts or contralateral vessels were used.

Venous patency is another key concern, as veins are more prone to proximal thrombosis in the zone of injury. Intraoperative confirmation using heparinized saline to ensure unrestricted flow is essential. When compromised, a more proximal vein and vein graft may be used. 21 Godina emphasized that recipient vein selection is the most critical determinant of flap outcome.

The worst limb salvage outcomes are typically associated with Gustilo–Anderson type IIIC injuries involving arterial disruption. These require emergent revascularization to salvage the limb. Stranix et al reported higher complication rates with an increasing number of injured vessels. 16 In cases with viable distal limbs but damaged vessels, use of a residual anterior tibial or posterior tibial artery is often advised. Older studies also considered retrograde flow through injured vessel as a viable option. 14 21

Several authors have associated posterior tibial vessel injury with higher amputation and flap failure rates. 25 26 The anterior tibial vessels, in contrast, are easier to access and may be turned up for use in proximal defects. 27 In our study, the anterior tibial artery was the most commonly used recipient vessel in both proximal and distal groups.

End-to-end anastomosis was the predominant technique (used in 70–90% of cases) across most large series, with a minority performed end-to-side. Our study followed a similar distribution.

Despite the denervation and vasodilation associated with free flaps, muscle flaps generally offer less resistance than fasciocutaneous flaps. 24 The New York group reported comparable outcomes using muscle flaps, 25 though higher failure rates were observed after takeback procedures. 28 In our study, flap type—fasciocutaneous, muscle, or composite—did not significantly affect outcomes.

A comparison of advantages of distal anastomosis and disadvantages of proximal is shown in Table 2 .

**Table 2:** Comparison between proximal and distal recipient vessel

Possible disadvantages of proximal anastomosis	Advantages of distal anastomosis
• Recipient vessels are located in deep planes • Extensive dissection • Time consuming • May need tunnelling or vein graft	• Recipient vessels are superficial • Ease of dissection • Less time consuming • Vein graft can be avoided

**Fig. 11:**
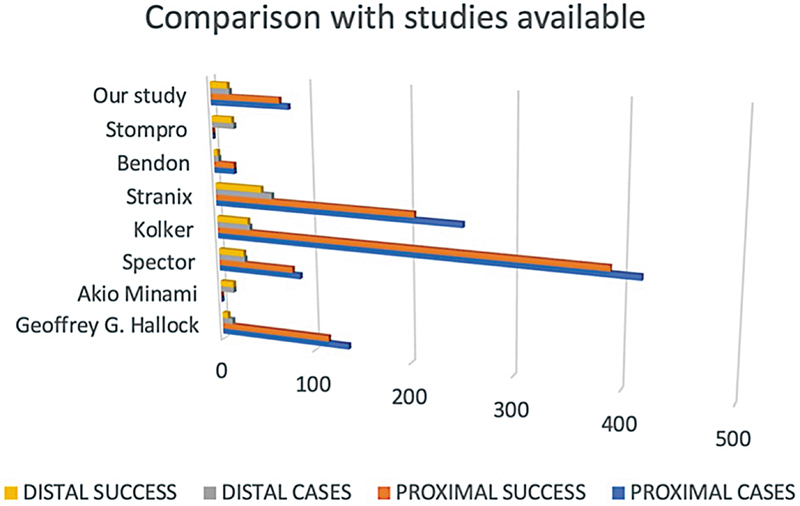
Comparison with other studies available.

A comparison of our findings with other published studies is presented in Table 3 and Fig. 11 .

**Table 3:** Our study compared with other available studies

Study	Proximal		Distal	
	Cases	Success	Cases	Success
Hallock 19	136	115 (84%)	11	6 (54%)
Akio Minami 29	a	a	14	14 (100%)
Spector et al 13	87	79 (91%)	28	27 (96%)
Kolker et al 4	416	388 (93%)	35	33 (94%)
Stranix et al 15	252	205 (90.7%)	60	49 (90.7%)
Bendon and Giele 18	21	21 (100%)	5	4 (80%)
Stompro and Stevenson 5	a	a	23	21 (91%)
Our study	81	72 (82.88%)	20	18 (90%)

### Limitations

This study is retrospective in nature, and available data were inherently skewed.

The choice of anastomosis site was ultimately influenced by multiple objective variables such as defect location, patient comorbidities, risk factors, vessel availability and patency, vessel size, and type of anastomosis—which may act as confounding factors.

Additionally, all distal anastomoses were performed by a single experienced surgeon, where proximal anastomoses were performed by multiple surgeons with varying levels of experience. This uneven distribution of surgical expertise and operator variability between the two groups represents a significant limitation of the study.

## Conclusion

Our study demonstrates that distal recipient vessel anastomosis in lower limb free flap reconstruction is a feasible and safe alternative to the proximal approach, with comparable flap survival and complication rates. Despite historical reluctance, distal vessels can be a valid option in appropriately selected cases. Further multicenter, prospective studies are needed to confirm these findings and inform clinical guidelines.
